# The SEB1741 Aptamer Is an Efficient Tool for Blocking CD4+ T Cell Activation Induced by Staphylococcal Enterotoxin B

**DOI:** 10.3390/molecules28083480

**Published:** 2023-04-14

**Authors:** Leslie Chavez-Galan, Andy Ruiz, Lucero A. Ramón-Luing, Alejandro Escamilla-Gutiérrez, Anahí Sánchez-Monciváis, Brenda Tecuatzi-Cadena, Karen Medina-Quero, María Guadalupe Córdova-Espinoza

**Affiliations:** 1 Laboratory of Integrative Immunology, Instituto Nacional de Enfermedades Respiratorias Ismael Cosío Villegas, Mexico City 14080, Mexico; 2 Laboratory of Medical Bacteriology, Department of Microbiology, Escuela Nacional de Ciencias Biológicas, Instituto Politécnico Nacional, Mexico City 11350, Mexico; 3 Hospital General “Dr. Gaudencio González Garza”, Centro Médico Nacional La Raza, Instituto Mexicano del Seguro Social IMSS, Mexico City 02990, Mexico; 4 Laboratory of Immunology, Escuela Militar de Graduados de Sanidad, SEDENA, Mexico City 11200, Mexico

**Keywords:** SEB, anti-SEB, SEB1741 aptamer, CD4+ T cells, inflammation

## Abstract

Staphylococcal enterotoxin B (SEB) is a protein produced by *Staphylococcus aureus*, which is toxic to humans. It is well known for its ability to stimulate the exacerbated activation of proinflammatory CD4+ T cells (Th1 profile), and in vitro studies have been conducted to understand its mechanism of action and its potential use as an immune therapy. However, the efficiency of the SEB1741 aptamer in blocking SEB has not been experimentally demonstrated. Methods: Enrichment CD4+ T cells were stimulated with SEB, and as a blocker, we used the SEB1741 aptamer, which was previously synthesised by an “in silico” analysis, showing high affinity and specificity to SEB. The efficiency of the SEB1741 aptamer in blocking CD4+ T cell activation was compared with that of an anti-SEB monoclonal antibody. Flow cytometry and Bio-Plex were used to evaluate the T-cell function. Results: In vitro, SEB induced the activation of CD4+ T cells and favoured a Th1 profile; however, the SEB1741 aptamer was highly efficient in decreasing the frequency of CD4+ T cells positive to ki-67 and CD69 cells, this means that proliferation and activation of CD4+ T cells was decreased. Moreover, the production of interleukin 2 (IL-2) and interferon-gamma (IFN-γ) was affected, suggesting that the Th1 profile is not present when the SEB1441 aptamer is used. Thus, the SEB1741 function was similar to that of anti-SEB. Conclusions: The SEB1741 aptamer is a valuable tool for blocking CD4+ T cell activation and the subsequent release of proinflammatory cytokines by SEB stimulation.

## 1. Introduction

*Staphylococcus aureus* (*S. aureus*) is a Gram-positive bacterium, and approximately 30% of the adult population is colonised by this bacterium. However, it plays a larger role than just being a commensal organism. *S. aureus* induces various human infections, including skin infections, upper respiratory tract infections, foodborne illnesses, and more severe illnesses, such as toxic shock syndrome [1,2,3]. Approximately 80% of *S. aureus* strains clinically isolated are enterotoxin producers [4], and staphylococcal enterotoxin B (SEB) is the main toxin produced by this bacterium. SEB is a superantigen (SAg) that activates T-cells in a non-specific manner, causing an exaggerated and potentially harmful immune response [3,4,5,6,7].

SAgs bind to the major histocompatibility complex class II (MHC-II) to stimulate the T-cell receptor (TCR) in many T cells, resulting in toxic shock syndrome due to the exacerbated release of proinflammatory cytokines [7,8,9,10]. In addition, a low concentration of SAgs in the bloodstream is enough to elevate toxic levels of proinflammatory cytokines, such as interleukin-2 (IL-2), interferon-gamma (IFN-γ), and tumour necrosis factor (TNF) [8,10,11,12].

The rapid onset of fever, organ failure, and, in some cases, death is a characteristic of toxic shock syndrome. Patient survival depends on the level of preexisting neutralising antibodies that prevent the binding of SAg/MHC-II, preventing the release of proinflammatory cytokines [10,13].

Treatment of toxic shock syndrome consists of antibiotics such as penicillin, cephalosporin, vancomycin, or methicillin, intravenous fluids, and immunoglobulin to neutralise the toxin; however, some of these drugs increase blood pressure, and cleaning and removal of infected tissue and antibiotic resistance may occur [14]. Thus, it is necessary to develop new treatments that focus on blocking T cell activation to avoid hyperinflammation in these patients.

The development of aptamers represents a therapeutic alternative with significant advantages; they are small RNA or DNA sequences, which by forming three-dimensional structures, can act as antibodies capable of binding to a given antigen with high recognition and affinity [15,16]. Thus, the design of a functional aptamer to inhibit the superantigenic activity of SEB could be an optimal alternative treatment. One study showed that in vitro, this aptamer significantly inhibited the production of proinflammatory cytokines, and in vivo, the survival rate was increased [15].

Recently, we reported the synthesis of an aptamer against SEB (hereafter called SEB1741), which by an “in silico” analysis, showed a prediction of high affinity and specificity in the binding site of SEB. The interactions are as follows: Cytosine 25-Lysine 57, Guanine 14-Glutamine 210, Adenine 30-Lysine 109 and 111, and Cytosine 23-Aspartic acid 108 [7]. The aptamer obtention was based on a prediction of binding affinity using three research algorithms (rDock, patchDock, and HDOCK). The binding affinity value between SEB and SEB1741 is only at a theoretical level, and algorithms provided the values “in silico.” Moreover, when the conventional AUTODOCK program calculates the dissociation constant (Kd) directly using small molecules with rotatable bonds, both molecules (SEB and SEB1741) have a considerable size, making them unpredictable to obtain the Kd.

Thus, this study aimed to evaluate the efficiency of the SEB1741 aptamer in blocking CD4+ T cell activation and the consequent proinflammatory cytokines released by SEB stimulation. Our data confirm experimentally that SEB1741 is efficient in blocking the interaction of SEB/CD4+ T cells, which helps regulate the production of IFN-γ but not that of TNF.

## 2. Results

### 2.1. The Aptamer SEB1741 Interacts with the Active Site of SEB

**Figure 1:**
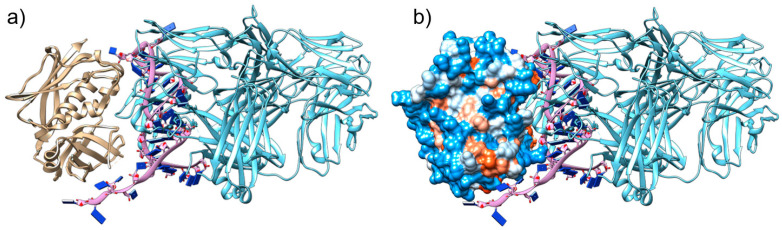
Molecular interactions between the tertiary structure of staphylococcal enterotoxin B (SEB), SEB1741, and monoclonal antibody (mAb) anti-SEB: (**a**) The beta-alpha helix SEB (brown) interacting with the hydrogen bonds of SEB1741 (purple) with Cytosine 25-Lysine 57, Guanine 14-Glutamine 210, Adenine 30-Lysine 109 and 111, and Cytosine 23-Aspartic acid 108; (**b**) SEB1741 (purple) and the mAb anti-SEB (light blue) interacting in the active site of the SEB hydrophobic region.

Previously, to select the best aptamer that could block the binding site of MHC-II molecules, we selected and characterised some aptamers “in silico” and identified SEB1741 as a potential blocking aptamer [7]. Figure 1a shows how SEB1741 interacts with the active site of SEB. Figure 1b shows the electrostatic surface of the SEB. According to the colour scale, red represents the hydrophobic character, and blue represents the hydrophilic character. The beta-alpha helix of SEB interacts by hydrogen bonds of SEB1741 with Cytosine 25-Lysine 57, Guanine 14-Glutamine 210, Adenine 30-Lysine 109 and 111, and Cytosine 23-Aspartic acid 108. In general, most strategies for blocking the immune response due to SEB SAg have focused on blocking the binding site that partially occupies the MHC-II binding site; however, in this case, the effort was to directly block the SEB binding site, suggesting that the SEB1741 aptamer could be an optimal option to avoid SEB/cell interactions.

### 2.2. The SEB1741 Aptamer Inhibits the CD4+ T-Cell Proliferation Induced by SEB

**Figure 2:**
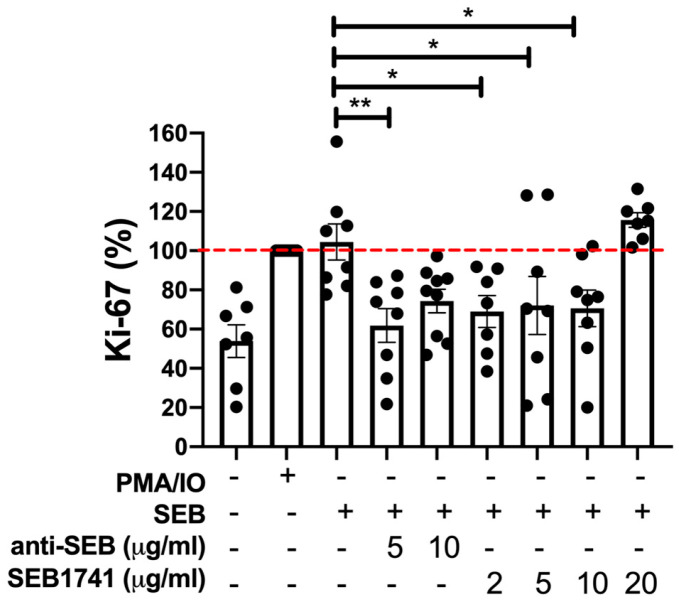
Staphylococcal enterotoxin B (SEB) increases the frequency of CD4+Ki-67+ T cells, but the SEB1741 aptamer inhibits this proliferation. The frequency of Ki-67 in human CD4+ T cells was assessed 48 h after stimulation with SEB, anti-SEB, or SEB1741 aptamer. PMA/IO was used as a positive control of activation, and it was normalised as 100% (dashed red line). Cell surface and intracellular staining were performed for the assessment using flow cytometry. n = six independent experiments, dots represent individual data, and bars represent mean value ± SEM. Differences between groups were analysed using the ANOVA and Dunnett’s tests as post hoc analyses. * *p* < 0.05, ** *p* < 0.01.

An increase in the rate of CD4+ cell proliferation may indicate an active immune response against diverse infections. Ki-67 is routinely evaluated as a proliferation marker [17,18]. Figure 2 shows that SEB induced a similar frequency of CD4+Ki-67+ T cells as the positive control (PMA/IO), confirming that SEB was efficient in activating CD4+ T cells. However, this frequency decreased when anti-SEB was used at 5 µg/mL (*p* < 0.01). Similarly, using the SEB1741 at concentrations of 2, 5, and 10 µg/mL, the frequency of CD4+Ki-67+ T cells was also decreased (*p* < 0.05).

It is essential to note that high concentrations of both anti-SEB (10 µg/mL) and the SEB1741 aptamer (10 µg/mL) were unable to block the proliferation of CD4+ T cells in response to SEB stimulus, suggesting that an excess of SEB blockers does not avoid the SAg function of SEB.

### 2.3. The SEB1741 Aptamer Decreases the CD4+ T-Cell Activation Induced by SEB

**Figure 3:**
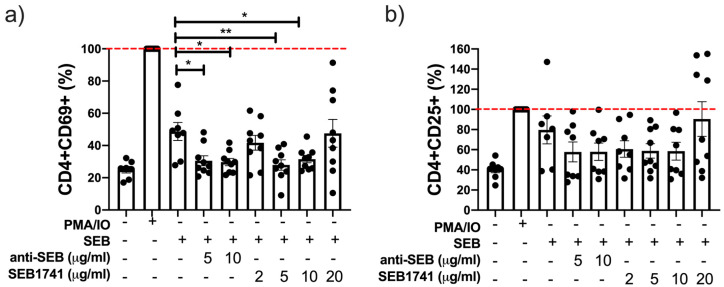
The frequency of CD4+CD69+ T cells, but not that of CD4+CD25+ T cells, is decreased by the SEB1741 aptamer. CD4+ T cells were recovered after 48 h of culture stimulated with staphylococcal enterotoxin B (SEB), anti-SEB, or SEB1741 aptamer, and PMA/IO was used as the activation control. Cell surface staining was performed and assessed using flow cytometry. The frequency of CD4+CD69+ (**a**) and CD4+CD25+ (**b**) was evaluated. n = six independent experiments, dots represent individual data, and bars represent mean value ± SEM. Differences between groups were analysed using the ANOVA and Dunnett’s tests as post hoc analyses. * *p* < 0.05, ** *p* < 0.01.

The expression of CD69 and CD25 on T cell surfaces is considered a classical marker of early and late activation, respectively [19]. Our data showed that SEB increased the frequency of CD4+CD69+ T cells after 48 h of culture, but not to the same level as PMA/IO (Figure 3a). Using the SEB1741 aptamer at 5 and 10 µg/mL was efficient in decreasing the frequency of the CD4+CD69+ T cells (*p* < 0.01 and *p* < 0.05, respectively). The anti-SEB also reduced the frequency of CD4+CD69+ T cells at 5 and 10 µg/mL (*p* < 0.05) (Figure 3a).

Regarding the frequency of CD4+CD25+ T cells or late activation cells, our data showed that SEB increased the frequency of this subpopulation, similar that the frequency induced with PMA/IO after 48 h of culture; however, neither anti-SEB nor SEB1741 decreased the frequency of CD4+CD25+ T cells (Figure 3b) significantly.

Together, these results suggest that the SEB1741 aptamer efficiently prevented the interaction of SEB/CD4+ T cells because of a decrease in activated CD4+ T cells.

### 2.4. Low Concentration of the SEB1741 Aptamer Decreases the Release of the Proinflammatory Cytokines IL-2 and IFN-γ but Not That of TNF

To confirm the efficiency of the SEB1741 aptamer in preventing the activation of CD4+ T cells, we evaluated the profile of pro- and anti-inflammatory cytokines in the supernatant of cultured CD4+ T cells. IL-2 is a cytokine that is produced as a response to a correct activation of T cells. In fact, CD25, the marker of cellular activation, is the receptor to IL-2, whereas IFN-γ and TNF are two of the most common proinflammatory cytokines [20,21].

**Figure 4:**
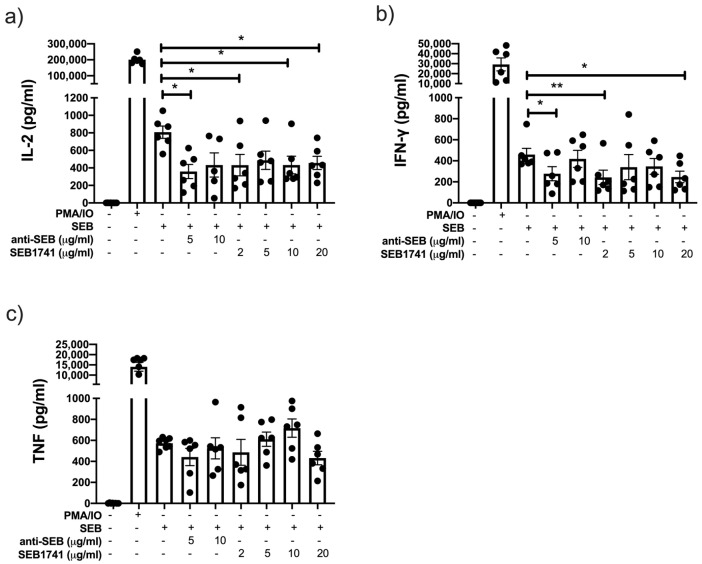
The SEB1741 aptamer efficiently decreases interleukin (IL)-2 and interferon-gamma (IFN)-γ production, but not tumour necrosis factor (TNF), in CD4+ T cells. IL-2 (**a**), IFN-γ (**b**), and TNF (**c**) levels were measured in supernatants collected from CD4+ T cells stimulated by staphylococcal enterotoxin B (SEB), anti-SEB, and SEB1741 aptamer by multiple cytokine assay. n = six independent experiments, dots represent individual data, and bars represent mean value ± SEM. Differences between groups were analysed using the ANOVA and Dunnett’s tests as post hoc analyses. * *p* < 0.05, ** *p* < 0.01.

Our in vitro system showed that SEB induced higher levels of IL-2, IFN-γ, and TNF than the unstimulated cells. However, these levels were lower than those of the positive control (PMA/IO), suggesting that after 48 h of culture, SEB efficiently activated and induced the release of proinflammatory cytokines by CD4+ T cells (Figure 4).

Thus, the SEB1741 aptamer at 2, 10, and 20 µg/mL decreased the release of IL-2 (*p* < 0.05), like 5 µg/mL of anti-SEB (Figure 4a). Furthermore, regarding IFN-γ levels, they were reduced using 2 and 20 µg/mL of SEB1741 (*p* < 0.01 and *p* < 0.05, respectively), whereas anti-SEB decreased IFN-γ levels at 5 µg/mL (*p* < 0.05) (Figure 4b). Finally, TNF was increased under SEB stimulus but was not downregulated with either anti-SEB or SEB1741 (Figure 4c).

Together, these results suggest that SEB efficiently induces the release of proinflammatory cytokines and that the SEB1741 aptamer efficiently decreases their release.

### 2.5. SEB Induces the Release of GM-CSF and IL-10 but Not That of Another Anti-Inflammatory Cytokine

The release of an anti-inflammatory profile was also evaluated in the supernatant of CD4+ T cells cultured under SEB stimulation.

**Figure 5:**
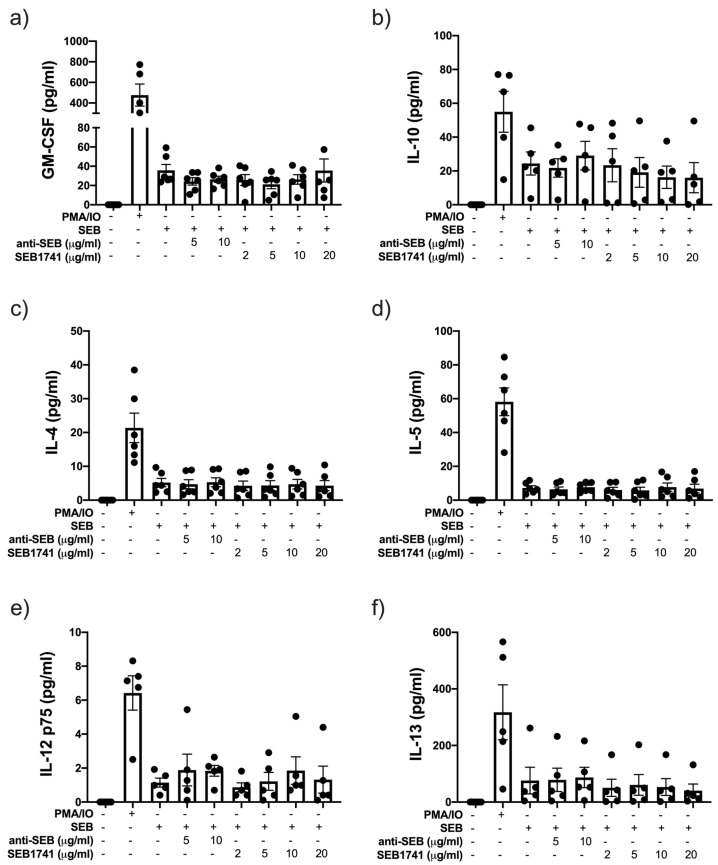
The addition of anti-staphylococcal enterotoxin B (SEB) and SEB1741 aptamers does not modify the production of GM-CSF (Granulocyte-macrophage colony-stimulating factor) interleukin (IL)-10, IL-4, IL-5, IL-12p75, and IL-13 in CD4+ T cells. The levels of GM-CSF (**a**), IL-10 (**b**), IL-4 (**c**), IL-5 (**d**), IL-12p75 (**e**), and IL-13 (**f**) were measured in supernatants collected from CD4+ T cells stimulated by SEB, anti-SEB and SEB1741 aptamer by multiple cytokine assay. n = six independent experiments, dots represent individual data, and bars represent mean value ± SEM.

Our data shows that PMA/IO is an optimal stimulus for inducing the release of a broad spectrum of cytokines. SEB induced higher levels of GM-CSF and IL-10 than in unstimulated cells, but neither anti-SEB nor SEB1741 inhibited this production (Figure 5a,b, respectively). Moreover, SEB did not induce the secretion of IL-4, IL-5, IL-12p75, or IL-13 (Figure 5c–f, respectively). Consequently, anti-SEB or SEB1741 did not show any effect.

## 3. Materials and Methods

### 3.1. Ethical Approval

All procedures performed in this study adhered to the principles stipulated in the Declaration of Helsinki. This study was approved by the Ethics Committee of the Military Central Hospital, Mexico City (protocol code: C.Inv.068). The healthy donors provided written informed consent for the use of their blood samples.

### 3.2. Peripheral Blood Mononuclear Cells (PBMCs)

Blood samples from six healthy donors (HD) were collected into EDTA tubes (BD Vacutainer, Franklin Lakes, NJ, USA). PBMCs were isolated within 1 h of blood collection and immediately used to isolate CD4+ T cells and develop cell cultures. PBMCs were isolated using standard Ficoll density gradient centrifugation (Lymphoprep Axis-Shield, Oslo, Norway), and cell viability was determined using trypan blue dye.

### 3.3. Isolation and Purification of CD4+ T Cells

CD4+ T cells were isolated by negative selection using the Human CD4+ T Cell Isolation Kit (Miltenyi Biotec, Germany) according to the manufacturer’s instructions. As a result, the enrichment of the CD4+ T cell fraction was  >96%, as determined by flow cytometry.

### 3.4. CD4+ T Cell Stimulation Assay with SEB

Isolated CD4+ T cells were plated in triplicate (2 × 10^5^ cells/200 μL), and SEB was added (1.7 μg/mL, Oxoid, Basingstoke, England) as the stimulus. The optimal concentration of SEB was established according to a previous standardisation, where CD4+ T cells were cultured for 48 h at 37 °C in 5% CO_2_ with SEB at 1.7 μg/mL and 8.5 μg/mL and the supernatant was recovered. TNF was quantified as an indirect marker of activation of CD4+ T cells.

Thus, the cell culture included one condition with SEB plus the monoclonal antibody (mAb) anti-SEB at 5 and 10 µg/mL (MyBioSource, San Diego, CA, USA) and another condition with the SEB1741 aptamer at concentrations 2, 5, 10, and 20 µg/mL (0.28 μM, 1.42 μM, 2.85 μM, and 5.7 μM, respectively). DNA aptamer (40 nt) was synthesised with a 5′-thiol group and HPLC (High-performance liquid chromatography) was purified by T4 Oligo^®^ (Guanajuato, México) at a concentration of 100 μM. In addition, a negative control (no stimulation) and positive control stimulated with phorbol myristate acetate (PMA) 50 nM–ionomycin (IO) 2 ng/μL (Sigma Aldrich, St. Louis, MO, USA) were included.

The cell culture was maintained for 48 h at 37 °C in 5% CO_2_. After incubation, the plate was centrifuged, and the supernatant was recovered and stored at −70 °C until it was used to measure the cytokine levels. However, the cells were prepared for flow cytometry.

### 3.5. Flow Cytometry

We used mAbs to evaluate the expression levels of CD4, CD25, CD69, and Ki-67 in cultured CD4+ T cells. To assess the expression of molecules on cell membranes, the cells were stained for 30 min at 4 °C in the dark. The cells were then fixed with 2% p-formaldehyde in phosphate-buffered saline (PBS, 0 mM sodium phosphate, 0.15 M sodium chloride, pH 7.2). The cells were washed to evaluate intracellular Ki-67 staining. Next, the cell pellet was suspended in a fixation/permeabilisation solution (eBioscience, Thermo Fisher, Waltham, MA, USA) at 4 °C, washed with permeabilisation buffer (eBioscience, Thermo Fisher, Waltham, MA, USA), and stained with Ki-67 for 30 min at 4 °C. Then, it was washed and analysed using flow cytometry. In addition, cells used for the Fluorescence Minus One (FMO) condition were stained and acquired in parallel to identify the background levels of staining.

Data were collected using a FACS Aria II (BD Biosciences, San Jose, CA, USA) and analysed using FlowJo v10.2 (FlowJo LLC, Inc., Ashland, OR, USA). For each case, 50,000 events are recorded. More details on the antibodies used are listed in Table A1.

### 3.6. Multiple Cytokine Assay in Supernatant Samples

A Bio-Plex Pro Human Cytokine Th1/Th2 Panel 9-Plex (Bio-Rad Laboratories, Richmond, CA, USA) was used. The level of Hu GM-CSF (Granulocyte-macrophage colony-stimulating factor), Hu IFN-γ, Hu IL-2, Hu IL-4, Hu IL-5, Hu IL-10, Hu IL-12(p70), Hu IL-13, and Hu TNF-a was measured in the supernatant of stimulated CD4+ T cell cultures, following the manufacturer’s instructions. Data were acquired using a Bio-Plex 200 System and analysed using Bio120 Plex Manager 6.1 software.

### 3.7. Statistical Analysis

Data are presented as mean ± standard error of the mean (SEM). Multiple comparisons were performed with the one-way ANOVA test with Dunnett’s multiple comparisons post hoc analysis. *p*-values < 0.05 were considered statistically significant (GraphPad Software V9, La Jolla, CA, USA).

## 4. Discussion

*S. aureus* is a highly ubiquitous pathogen with various virulence factors that have helped develop immune evasion strategies. Within the family of staphylococcal enterotoxins (SEs), in addition to SEB, toxic shock syndrome toxin 1 (TSST-1) is included, and it has been reported to be highly associated with SEB cases [22]. Staphylococcal toxic shock syndrome (TSS) has a mortality rate of 50–60%, depending on the severity of the clinical case [23].

The first epidemic of staphylococcal toxic shock syndrome was reported in 1980; subsequently, in the 1990s, the incidence of TSS associated with menstruation and the proportion of non-menstrual cases increased. Non-menstrual TSS has been reported mostly in association with any primary staphylococcal infection following surgery, mainly in cases where there has been disruption of mechanical barriers or placement of a medical device [24]. Many of these reasons prompted several studies to develop toxoid vaccines or peptides that mimic regions in the structure of superantigens and antibodies produced to block their activity [25].

SEB is an SAg produced by *S. aureus* that can interact with the active site of the major histocompatibility complex class II (MHC-II) to activate CD4+ T cells through the T-cell receptor (TCR) [3,26]. There is then massive activation of these T cells, which are a subset of cells that help coordinate the immune response and lead to the excessive release of proinflammatory cytokines [27]. This means that SAg binds outside the complementarity-determining regions (CDRs) of the TCR, causing the cross-linking of MHC-II/TCR. The magnitude of the hyperactivation status induced by an SAg could be dimensional, considering that in a normal adaptive immune response, only around 0.0001% of T cells are activated, whereas SAg activates up to 30% of the T-cell pool [28].

One of the consequences of massive T-cell activation is an increase in leukocyte recruitment, which could favour the survival of *S. aureus* within neutrophils and macrophages due to hyperactivation, eventually leading to T-cell anergy and cell death [29].

Currently, there are no approved vaccines or specific drugs for treating SEB-induced illnesses. Moreover, the use of mAbs has been suggested to block the action of SEB [30,31,32,33]. However, the production of anti-SEB mAbs has disadvantages such as high manufacturing costs, variable specificity, and difficulty in production or storage. Recently, the possibility of using a single-chain biparatopic bispecific antibody to target SEB and allosterically prevent TCR binding has been explored. However, this has been performed only as a computational analysis [33]. Antibodies against LXY8, Ig121, c19F1, and 20B1 have been used in experimental models [34,35,36]. Currently, they are not used in humans, indicating the need to focus on the development of therapeutic alternatives.

Thus, the search has not been limited to blocking the interaction between SEB and TCR. The use of anti-inflammatory drugs, such as sulfasalazine [37], or immunosuppressive drugs, such as rapamycin [38], could be useful in decreasing the excessive inflammatory status. Although they are alternatives to treat the symptoms, they do not prevent massive hyperactivation of T cells.

The development of aptamers has also been proposed to improve treatment schemes [15] and could be an optimal tool because aptamers have high specificity and sensitivity, similar to antibodies. Over the last 25 years, 18 nucleic acid-based therapeutics have been approved for the treatment of various diseases. Compared to mAbs, aptamers have advantages such as stability for long-term storage, simplicity of synthesis and function, and low immunogenicity [39,40].

In this context, previously, we reported the selection “in silico” of the SEB1741 aptamer using a strategy similar to “in vitro” SELEX, including the negative and positive rounds to select the best aptamer with the higher values of interaction with SEB and no other toxins [7]. This approach allowed us to find the SEB1741, and based on the “in silico” analysis, we proposed that it could inhibit the CD4+ T cell activation to regulate the inflammatory status induced by SEB. Here, we showed that the SEB1741 aptamer decreased the percentage of Ki-67 cells and IL-2, suggesting that these molecules inhibit T-cell activation.

Under diverse pathologies, regulating the excessive activation of CD4+ T cells is necessary to maintain homeostasis; therefore, targets have been studied to limit CD4+ T cell activation [41,42]. This study showed that CD4+ T cells treated with the SEB1741 aptamer showed decreased SEB-induced activation and the release of proinflammatory cytokines, specifically IL-2 and IFN-γ, but not TNF. This suggests that SEB1741 is efficient in blocking the IFN-γ-dependent inflammatory pathway. IFN-γ production is one of the most important characteristics of diseases with massive inflammation. Moreover, it has recently been reported that SAg induces excessive IFN-γ levels, which allows the replication of bacteria, such as *S. aureus*, within macrophages, promoting bacterial survival by manipulating the immune response [8].

TNF is a proinflammatory cytokine that activates cell death pathways, mainly via the TNF receptor 1. However, TNF was not decreased by either SEB1741 or anti-SEB, suggesting that this pathway was not inhibited, even when using the antibody. The regulation of TNF production induced by SEB stimuli probably requires the use of molecules, such as drugs, whose functions are performed inside the cell. Bisdemethoxycurcumin has been reported as a potential natural antibacterial agent to decrease the TNF induced by SEB or staphylococcal enterotoxin A (SEA) [43]. Similarly, black ginger has been proposed as a promising natural compound for reducing TNF production, even in methicillin-resistant *S. aureus* [44].

Evidence suggests that SEB blockers do not prevent TNF production; apparently, this pathway is regulated differently from IFN-γ production, at least by SEB stimulus. Thus, the search for therapies that block TNF production must be addressed simultaneously. Although cytotoxic cells were not evaluated in this study, it is a new open question because we do not know whether SEB1741 also inhibits the binding of SEB to CD8+ T cells or if it blocks only the interaction between SEB/CD4+ T cells.

Moreover, it is worth noting that an excess of aptamers can lose their ability to inhibit proliferation, similar to what is observed with an excess of anti-SEBs. Research has shown that an excess of antibodies or antigens can interfere with signalling for T cell activation and result in a lack of IL-2 production and phosphorylation of signalling molecules [45]. Thus, determining the optimal concentration of aptamers is critical to effectively assay the function of a blocking antibody.

Our study has some limitations. For instance, we did not experimentally evaluate other aptamers in parallel to confirm the high specificity of SEB1741. Nonetheless, as previously reported, the aptamer library we used to identify the best aptamers for SEB, cholera toxin, and botulinum toxin displayed high specificity to their respective toxins “in silico” and did not exhibit cross-reactivity [7]. Additionally, further studies should be conducted “in vivo” to evaluate inflammation and survival before suggesting the use of SEB1741 as an alternative treatment for toxic shock syndrome.

Despite the limitations in our study, these data strongly suggest that SEB1741 has the potential to be an optimal tool for preventing TSS through an excessive inflammatory response mediated by IFN-γ produced by CD4+ T cells. Like antibodies, aptamers bind their targets by folding them into a specific three-dimensional conformation dictated by the nucleic acid sequence. In addition to aptamers having a higher affinity, they display a lower Kd and thus bind better to the target molecule [46].

These features represent a combination of the best features of small molecules and antibodies, providing great potential for numerous therapeutic applications, including the inhibition of proinflammatory proteins. Additionally, from the perspective of the pharmaceutical industry, aptamer production could represent a lower cost than antibody production.

## 5. Conclusions

These results suggest that the SEB1741 aptamer has therapeutic potential as an alternative to antibodies and other protein inhibitors currently used to treat SAg-mediated diseases. Interestingly, this aptamer specifically blocks the exacerbated production of IFN-γ but not that of other proinflammatory cytokines, such as TNF. Moreover, further studies are needed to evaluate its efficacy and long-term safety in vivo.

## Data Availability

The authors confirm that the raw data used to support the conclusions of this study are included in the manuscript. The corresponding author will provide more details upon request from any qualified researcher.

## Appendix A

**Table A1:** Information on the antibodies used in this study.

Antibody	Company	Clone	Cat. Number
Brilliant Violet 421™ anti-human Ki-67	BioLegend	Ki67	B269687
PE/Cyanine7 anti-human CD4	BioLegend	OKT4	B159354
PE-A anti-human CD25	BD Pharmingen	BC96	3235775
Brilliant Violet 510™ anti-human CD69	BioLegend	FN50	B322621
